# Cross-border outbreak of *Yersinia enterocolitica* O3 associated with imported fresh spinach, Sweden and Denmark, March 2019

**DOI:** 10.2807/1560-7917.ES.2019.24.24.1900368

**Published:** 2019-06-13

**Authors:** Laura Espenhain, Maximilian Riess, Luise Müller, Soledad Colombe, Steen Ethelberg, Eva Litrup, Cecilia Jernberg, Sharon Kühlmann-Berenzon, Mats Lindblad, Nikolas Kühn Hove, Mia Torpdahl, Marie Jansson Mörk

**Affiliations:** 1 Statens Serum Institut, Copenhagen, Denmark; 2 These authors contributed equally to the work and share first authorship; 3 Public Health Agency of Sweden, Solna, Sweden; 4 European Public Health Microbiology Training Programme (EUPHEM), European Centre for Disease Prevention and Control (ECDC), Solna, Sweden; 5 European Programme for Intervention Epidemiology Training (EPIET), European Centre for Disease Prevention and Control (ECDC), Solna, Sweden; 6 Department of Public Health, Faculty of Health and Medical Sciences, University of Copenhagen, Copenhagen, Denmark; 7 National Food Agency, Uppsala, Sweden; 8 Danish Veterinary and Food Administration, Copenhagen, Denmark

**Keywords:** Disease Outbreaks, Yersinia Infections, Epidemiology, Intersectoral Collaboration, Whole Genome Sequencing, WGS

## Abstract

In April 2019, a cross-border outbreak of *Yersinia entercolitica* O3 was identified in Sweden and Denmark and confirmed using whole genome sequencing. Close cross-border collaboration with representatives from human and food authorities helped direct resources and investigations. Combined epidemiological and trace-back investigations pointed to imported fresh spinach as the outbreak vehicle and highlight that other vehicles of *Y. enterocolitica* outbreaks than pork should be considered.

Here we describe the results of the investigation of a cross-border outbreak with *Yersinia* *enterocolitica* associated with imported fresh spinach. The aim is to highlight the importance of early communication of signals, rapid access to whole genome sequencing (WGS) and the value of collaboration between public health and food agencies across borders. We also stress the necessity to increase awareness about vehicles of *Y. enterocolitica* outbreaks other than pork.

## Identification of the outbreak

In early April 2019, the Public Health Agency of Sweden (PHAS) and Statens Serum Institut (SSI) independently noted an increase in *Y. enterocolitica* and *Y. enterocolitica* O3 biotype 4 cases as part of routine surveillance. In Sweden, WGS revealed that isolates were closely related on a genetic level. On 10 April, PHAS contacted and shared a representative outbreak sequence with public health institutes in Denmark, Finland and Norway to inquire whether a matching cluster had also been observed. SSI reported a similar signal and started sequencing their *Y. enterocolitica* O3 biotype 4 isolates. Swedish and Danish sequences were compared and found to be of sequence type (ST) 18 and genetically closely related. A cross-border outbreak was declared on 24 April 2019.

## International response

An urgent inquiry (UI-554) for cases in other European countries was posted on the European Centre for Disease prevention and Control (ECDC) Epidemic Intelligence Information System (EPIS) and a notification was sent through the European Commission’s Early Warning and Response System (EWRS) on 26 April 2019. Two representative outbreak sequences from Denmark were deposited to the European Nt Archive (https://www.ebi.ac.uk/ena), number ERR3293974 (1903T46767) and ERR3293975 (1903H32600).

## Case definition

For the outbreak investigation, a confirmed case was defined as an individual residing in Sweden or Denmark with laboratory-confirmed *Y. enterocolitica* O3 sequenced at SSI and PHAS and found to be ST18 and to belong to the specific genetic cluster, with a reporting date between 1 March and 21 May 2019.

To anticipate future confirmed cases during the outbreak, a probable case was, in Denmark, defined as *Y. enterocolitica* O3 and, in Sweden, as *Y. enterocolitica* O3 or *Y. enterocolitica* with unknown serotype.

## Case finding

No active case finding for people with relevant symptoms was carried out.

## Descriptive epidemiology

**Figure 1:**
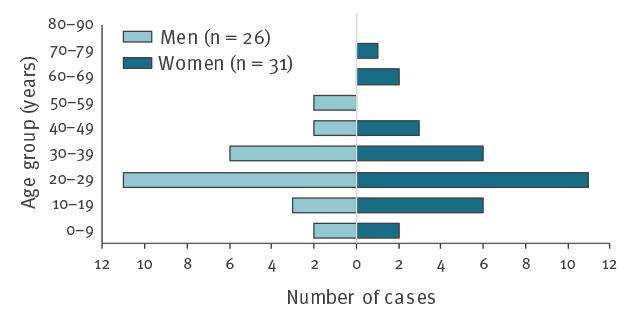
Age and sex distribution of cases of *Yersinia* O3 biotype 4 ST18 infection, Denmark and Sweden, March 2019 (n = 57)

**Figure 2:**
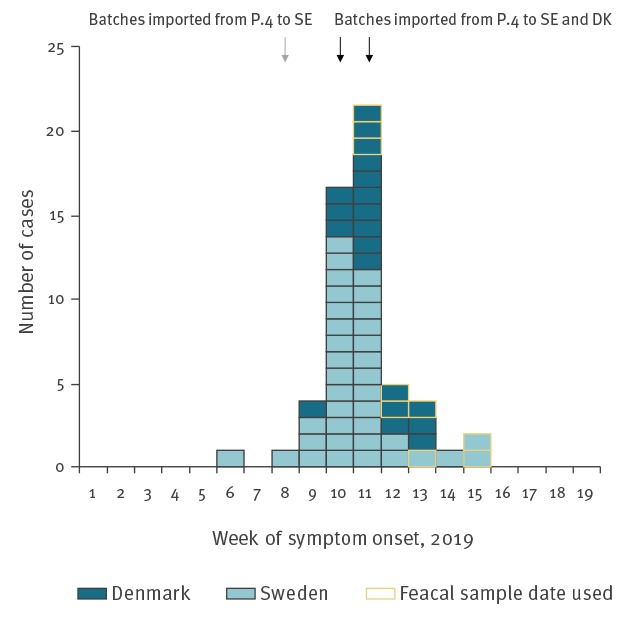
Number of confirmed outbreak cases by country and week of onset of symptoms^a^, Sweden and Denmark, 1 January–12 May 2019 (n = 57)

DK: Denmark; SE: Sweden.

^a^ If the week of onset was unknown, then week of faecal sample (orange outline) was used.

A total of 57 cases were confirmed, 37 from Sweden and 20 from Denmark; 30 cases were aged 20–35 years old, the overall age range of cases was 2–74 years and 31 were female (Figure 1). Cases resided in all five Danish regions and in 13 of 21 Swedish counties. Date of onset was known for 48 cases and ranged from 10 February to 3 April, with a peak in weeks 10 and 11 (4–16 March) (Figure 2). Date of sampling ranged from 8 March to 11 April and peaked in week 12 (18–24 March).

ST: sequence type.

Black arrows indicate the week of import batches from the common producer P.4 to both Sweden and Denmark. The grey arrow indicates the week of import for batches from producer P.4 to Sweden.

## Epidemiological investigation

### Hypothesis generation

Cases were interviewed using hypothesis-generating questionnaires about food consumption 7 and 10 days before disease onset in Denmark and Sweden, respectively. Epidemiologists from SSI interviewed cases in Denmark and the local county medical officers interviewed cases in Sweden. Data from 16 of 20 Danish cases and 31 of 37 Swedish cases was used.

Many of the Danish cases had a diet consisting of a lot of vegetables and little or no meat, two cases were vegan. The majority of Danish cases (13/16) reported shopping in retail chain A, with the remainder reporting having shopped in retail chain B, belonging to the same retail group (X) as A. No unusual food consumption pattern was noted for the Swedish cases, 19 of 24 cases regularly shopped in retail chain C.

### Case–control studies

Independent case–control studies were carried out in each country to test hypotheses of frequently consumed food items being the vehicle of the outbreak. In Denmark, spinach was suspected as the source as all interviewed cases (16/16) had eaten fresh spinach the week before onset of symptoms.

In Denmark, three controls were selected from the civil registration register and matched to each case by sex, municipality of residence and date of birth (± 90 days). In Sweden eight controls were selected from a national random pool of controls (n = 5,900) available at PHAS; the controls were matched to each case by sex and ± 5 years of age and matched on county of residence or neighbouring counties. Controls were excluded if they had travelled or reported having had symptoms compatible with *Yersinia* infection before the interview.

All Danish cases (16/16) had eaten spinach compared with 6 of 45 controls (13%). Spinach, bagged salad mix, tomatoes, raspberries and raw carrots were found to be borderline statistically significant (p < 0.1) in univariable analyses using Firth logistic regression (firthlogit Stata version 14.2, Stata Corp., College Station, Texas, United States) to deal with the zero unexposed among cases. When the five food items were included in a multivariable analysis, adjusted for age and sex, only spinach remained significant (Table).

**Table:** Univariable and multivariable results of the Danish and Swedish case–control studies, adjusted for age and sex, sorted by most common exposure, Sweden^a^ and Denmark^b^, March 2019

Food exposure	Denmark						Sweden					
	Cases exposed		Univariable^c^		Multivariable^c^		Cases exposed		Univariable^c^		Multivariable^c^	
	Proportion n/N	%	aOR	95% CI	aOR	95% CI	Proportion n/N	%	aOR	95% CI	aOR	95% CI
Fresh spinach	16/16	100	164	(9.5–2,800)	113	(3.7–3,400)	20/29	69	1.4	(0.60–3.4)	1.4	(0.53–3.7)
Tomatoes	15/16	94	5.4	(0.91–32)	3.6	(0.24–56)	25/29	86	0.3	(0.10–1.0)	NI	NI
Raw carrots	13/16	81	3.1	(0.84–11)	1.5	(0.08–32)	20/29	69	0.7	(0.28–1.6)	NI	NI
Bagged salad mix	12/16	75	6.0	(1.7–21)	2.0	(0.13–31)	H	NA	NA	NA	NA	NA
Raspberries	10/16	63	5.0	(1.5–17)	7.4	(0.60–91)	9/28	32	1.8	(0.78–4.3)	1.4	(0.49–4.2)
Eating at a canteen	7/16	44	C	NA	NA	NA	15/28	54	1.6	(0.70–3.8)	1.9	(0.40–2.4)
Mixed minced meat	2/14	14	C	NA	NA	NA	16/28	57	1.5	(0.66–3.2)	1.0	(0.40–2.4)
Raisins	H	NA	NA	NA	NA	NA	13/29	45	1.6	(0.73–3.5)	1.8	(0.75–4.4)
Blueberries	H	NA	NA	NA	NA	NA	8/29	28	1.9	(0.78–4.5)	1.3	(0.41–3.9)
Baby corn	H	NA	NA	NA	NA	NA	7/29	24	1.6	(0.62–4.0)	NI	NI
Kale	H	NA	NA	NA	NA	NA	7/29	24	1.5	(0.61–3.8)	1.9	(0.69–5.1)
Ready-to-eat salad bowl	H	NA	NA	NA	NA	NA	7/30	23	1.5	(0.61–3.8)	NI	NI

aOR: adjusted odds ratio; C: not included in case–control study; CI: confidence interval; H: not asked in the hypothesis-generating questionnaire; NA: not available; NI: not included in final multivariable model.

^a^ 30 cases and 405 controls were included.

^b^ 16 cases and 45 controls were included.

^c^ Adjusted for age and sex.

The Swedish case–control study also pointed towards a vegetable or fruit product and not pork, but no statistically significant food items were found in either univariable or multivariable analyses. Among Swedish cases, 20 of 29 (69%) had eaten spinach compared with 211 of 393 (54%) of controls (adjusted odd ratio = 1.4, (95% confidence interval: 0.5–3.7) by multivariable analysis) (Table).

## Trace-back investigation of spinach

The Danish Food and Veterinary Agency (DFVA) and the Swedish National Food Agency (NFA) conducted a trace-back investigation for fresh spinach sold in retail chain A-C from the point of sale back to the producers.

**Figure 3:**
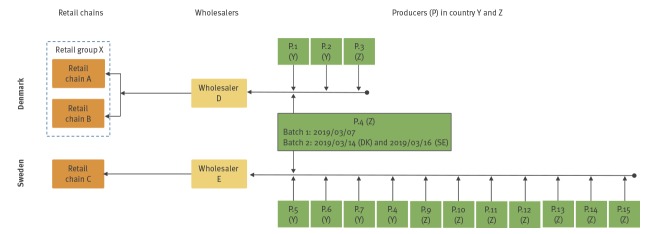
Overview of the trace-back of fresh spinach sold in retail chains A–C, Denmark and Sweden, March 2019

In Sweden, one wholesaler (E, Figure 3) delivers all fresh spinach sold by retail chain C under their private label. In Denmark, DVFA investigations showed that one wholesaler (D, Figure 3) was supplying fresh spinach to retail group X during weeks 9–10. Tracing back deliveries of fresh spinach (end of February to the middle of March) to wholesaler D in Denmark and wholesaler E in Sweden, showed that all imported spinach came from two countries within Europe and that the two wholesalers had imported spinach from one common producer (P.4, Figure 3). Additional investigation showed that two specific batches of spinach had been imported to both countries from producer 4: one batch on 7 March and a second batch imported on 14 March and 16 March to Denmark and Sweden, respectively, the final product was in the Swedish retail stores a maximum of three days after import. The same producer, P.4, had also delivered fresh spinach to the Swedish wholesaler on 19 and 22 February. Thus, the results of the trace-back investigation point to fresh spinach from P.4 as a possible common source of the outbreak. No spinach from the implicated batches was available for testing and no recall was performed.

Consumer purchase data was collected from Danish patients [1] where feasible. Receipts from purchases made in retail chain A from three Danish cases were collected and bagged fresh spinach products were identified for all three.

## Discussion and conclusion

Here we report a cross-border outbreak of *Y. enterocolitica* confirmed by WGS and investigated through cross-border collaboration between Sweden and Denmark. Typical symptoms of yersiniosis include abdominal pain, which can mimic appendicitis, and self-limiting acute febrile diarrhoea. *Y. entercoloitica* infections lead to sequelae such as reactive arthritis and erythema nodosum in around 10% of cases [2]. Two case–control studies were performed. The Swedish study pointed towards vegetable or fruit products, but no food item was consumed more by cases than controls. The Danish study pointed at fresh spinach as the vehicle of the outbreak. Trace-back investigation corroborated this finding by linking the spinach sold in Denmark and Sweden to a single common producer and two specific batches. No other European countries reported having cases connected to the outbreak. The distribution of the *Y. enterocolitica* cases within both countries and the short period of illness onset (2 weeks) further support that the source of infection was a product that was widely distributed and had a short shelf life or was available only for a short period of time. Two Danish and 11 Swedish cases reported their date of onset of symptoms as being earlier than the date of import of the first common batch. However, all cases had a faecal sample date after the first import date. Whether the early cases are explained by incomplete recall, illness due to a different pathogen before yersiniosis or for the Swedish cases potential contamination of earlier imported lots from the same producer, is unknown.

Pathogenic *Y. enterocolitica* is found in the throat and intestine of pigs and transmission is usually associated with eating raw or undercooked pork [3,4]. This outbreak, however, suggests that other sources of *Y. enterocolitica* outbreaks may play a role. Other food items like spinach could be contaminated with *Y. enterocolitica* through contaminated irrigation water, fertilizer or other environmental sources, for example. From 2011 to 2018, three outbreaks linked to imported ready-to-eat salad mix products have been described in Norway [5-8]. In 2015, Gupta et al. [9] listed a number of outbreaks of *Y. enterocolitica* linked to contamination of fresh vegetables including salads. Pathogen bacterial contamination including *Y. enterocolitica* has been described in leafy green vegetables and pre-packed salad mix [10,11].

*Y. enterocolitica* is a common cause of bacterial diarrhoeal disease in Sweden and Denmark, with 2.3 and 4.9 cases per 100,000 inhabitants in 2016, respectively [12]. In both Denmark and Sweden, *Y. enterocolitica* isolates are only typed using WGS in case of suspicion of outbreaks. Since the majority of isolates belong to serotype 3, easy access to WGS in case of outbreak-signals is crucial for outbreak confirmation and investigation.

This cross-border outbreak would not have been identified without good communication lines between Sweden and Denmark, access to WGS and early sharing of sequence and epidemiological information. The close cross-border collaboration with representatives from both the human and food authorities helped direct resources and investigations so that the identification of the likely source was possible. The implication of spinach reinforces the need for improved control measures in the production chain for fresh produce. Currently wholesalers routinely sample pre-cut vegetables for analyses of *Salmonella*, *Escherichia coli* and *Listeria monocytogenes* according to microbiological criteria in European Commission Regulation (EC) No 2073/2005 [13], but normally not for other pathogens. The findings also highlight that fresh produce should be considered as possible sources of *Y. enterocolitica* outbreaks in the future.

## Status as at 12 June 2019

As at 12 June 2019, no new outbreak cases have been reported. No recall was performed and no testing of subsequent batches was done as spinach from the implicated batches was not on the marked and no more cases were reported at the time of the epidemiological results. The DFVA posted a Rapid Alert System for Food and Feed (RASFF) notification on 12 June to inform the exporting country in order for them to follow up with the producer.
